# Tracheal Nodular Fasciitis Presenting with Stridor: A Rare Case Report

**DOI:** 10.22038/ijorl.2025.85764.3885

**Published:** 2026

**Authors:** Manaswini Mallick, Debasmita Rath, Alok Das, Aparna Mohanty, Krishna Sai Sharan, Swagatika Samal

**Affiliations:** 1 *Department of ENT, IMS & SUM Hospital, Bhubaneswar, India.*; 2 *Department of Pediatrics, IMS & SUM Hospital, Bhubaneswar, India.*; 3 *Department of Pathology, IMS & SUM Hospital, Bhubaneswar, India.*

**Keywords:** Tracheal obstruction, Nodular fasciitis, Immunohistochemistry

## Abstract

**Introduction:**

Nodular fasciitis (NF) is a benign, rapidly growing, non-neoplastic myofibroblastic lesion of the subcutaneous tissue affecting various anatomical sites throughout the body. NF of the trachea is rare, and its occurrence in the pediatric age group is highly uncommon in clinical practice.

**Case Report:**

2-year-old female child who presented to the emergency department with severe stridor causing critical airway obstruction. The mass was successfully excised via an emergent surgical procedure, and the definitive diagnosis of nodular fasciitis was established through histopathology examination, which revealed proliferation of spindle-shaped myofibroblasts in a myxoid stroma without features of malignancy.

**Conclusion:**

Early resuscitation is essential to save the patient's life, and histopathology confirms the diagnosis.

## Introduction

Nodular fasciitis (NF) is characterized as a non-malignant, proliferative, reactive lesion composed of fibroblasts and myofibroblasts (1).

It is found most often in the extremities and trunk. Less frequently, it involves the head and neck, including eyelids, face, neck, and oral cavity. In rare instances, NF has been reported in sites such as the urinary bladder, mammary gland, and vocal cords. 

A review of existing literature reveals that only a limited number of cases involving the larynx and the involvement of the trachea presenting with life-threatening stridor are yet to be documented. Here, we present a rare case of NF causing significant airway obstruction in a child, and discuss its diagnostic challenges and therapeutic management.

## Case Report

A 2-year-old girl presented to the emergency department with an acute onset of severe stridor. On examination, she exhibited marked severe respiratory distress with a significantly reduced oxygen saturation level. A critical care team was immediately activated, and the patient was resuscitated with life-saving interventions. The patient was intubated with the smallest available endotracheal tube, and an urgent ENT consultation was requested for further evaluation. According to the parents, the patient had a history of recurrent cough and dyspnea over the previous three months and was treated for the same with temporary relief. Her respiratory symptoms have gradually worsened for the past 2 months. 

There was no history of foreign body aspiration or hemoptysis with the patient. The patient was intubated as an emergency measure and subsequently referred to the ENT Department for bronchoscopic evaluation. Fiberoptic bronchoscopy was performed through the endotracheal tube, and an ill-defined lesion was detected in the trachea above the carina. 

**Fig 1:**
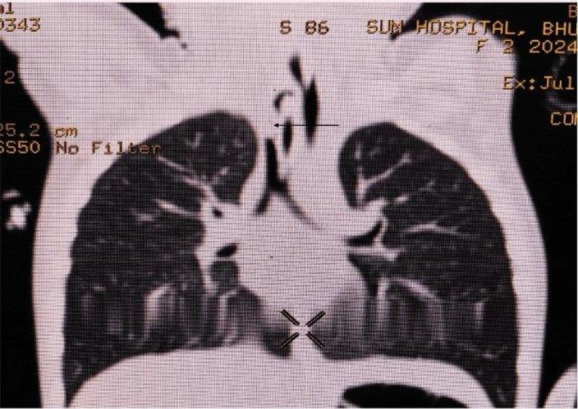
HRCT thorax shows a well-defined oval-shaped hypodense submucosal lesion of size 9.4x7.5x12. 5 mm arising from the right lateral wall of trachea, approximately 2.2 cm proximal to the carina (indicated by red arrow) with endotracheal tube in situ (indicated by green arrow)

A non-contrast computed tomography scan was subsequently performed to evaluate the tracheal lesion/foreign body further. The scan revealed a space-occupying lesion located in the trachea, approximately 2 cm above the bifurcation, measuring 1.5 x 1 cm, along with pneumonic consolidation in the right lower lobe (Fig. 1). 

**Fig 2:**
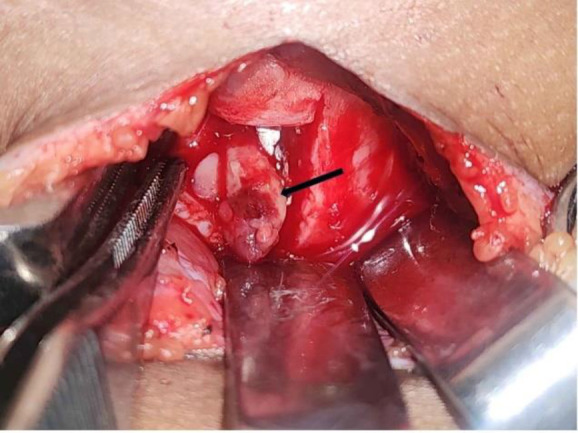
The tumor was found approximately 2 cm above the carina, almost occluding the tracheal lumen

The patient was then taken up for urgent fiberoptic bronchoscopy under general anesthesia. A whitish, firm mass was visualized approximately 2 cm above the carina, obstructing about 75% of the tracheal lumen (Fig 2). 

**Fig 3:**
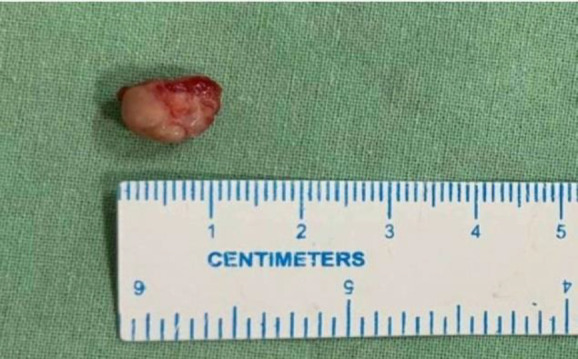
Gross examination, the lesion shows a polypoid tumour measuring 1.2 cm in greatest dimension with focal ulceration.

The lesion had surface erosion with overlying exudate. After a thorough discussion with the parents and obtaining informed consent, the patient was planned for definitive surgery. A low tracheotomy was performed due to the distal location of the trachea mass. The mass was visualized and removed through the tracheotomy window. No tracheostomy tube was inserted during the surgical procedure. A smooth, rounded mass was identified in the lower trachea, pedicled to the right tracheal wall. The mass was excised entirely with direct endoscopic visualization using intermittent apnea with jet ventilation. Hemostasis was achieved by using the Coblator device. The tracheotomy was closed after complete hemostasis. The patient was intubated postoperatively and observed in the pediatric intensive care unit for 7 days until the chest pathology improved, and after which she was extubated. Later, it was sent for histopathological examination. The mass was polypoid, soft, and friable on gross examination (Fig 3).

Microscopically, it was lined by nonkeratinized stratified squamous epithelium with focal ulceration over the surface. The subepithelial tissue revealed a spindle cell tumor with alternating hypo and hypercellular areas. 

The tumor cells were arranged in short fascicles and a storiform pattern. High magnification showed mild nuclear pleomorphism and occasional mitotic activity. Immunohistochemistry demonstrated diffuse smooth muscle actin (SMA) positivity, focal S100 positivity, and negative staining for Desmin, SOX10, and CD34. 

**Fig 4:**
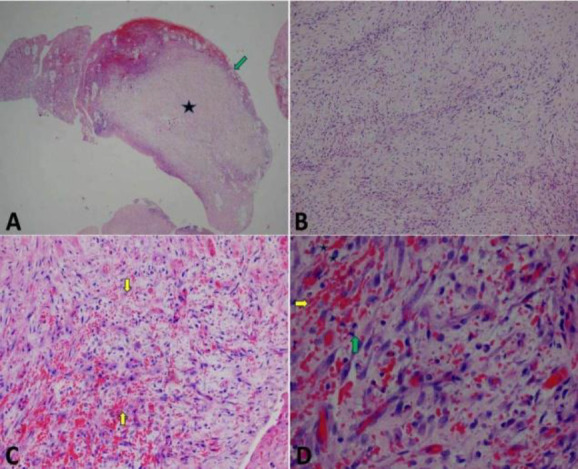
**A;** Microsection shows a polypoid tumor lined by nonkeratinized stratified squamous epithelium (arrow) with marked ulceration covered with hemorrhage and exudate; subepithelium shows spindle cell tumor (asteric). (H&E, 20x), **B;** Microsection shows the tumor comprising spindle cells arranged in short fascicles, intersecting fascicles and storiform patterns. (H&E, 100x), **C;** Microsection shows the tumor comprising of plump spindle cells/ myofibroblasts interspersed by thin-walled capillaries with extravasation of RBCs (indicated by yellow arrow (indicated by green arrows) (H&E, 200x), **D;** Microsection shows the tumor comprising of plump spindle cells/ myofibroblasts interspersed by thin-walled capillaries with extravasation of RBCs (indicated by yellow arrow). Occasional mitotic figures identified (indicated by green arrow) (H&E, 400x).

**Fig 5:**
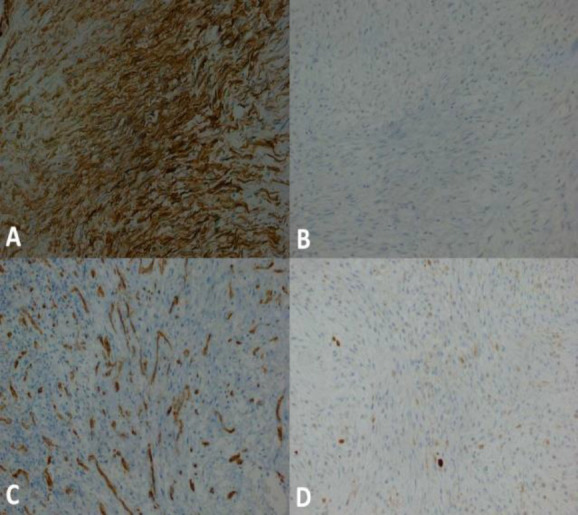
**A; **The tumor cells show diffuse immunopositivity for SMA (IHC, 200x); **B;** The tumor cells are immunonegative for Desmine (IHC, 200x), **C;** The tumor cells are immunonegative for CD34 (black arrow), while CD34 highlight the endothelial cells of capillaries (green arrow) (IHC, 200x), **D;** The tumor cells show focal weak positivity for S100 (IHC, 200x).

The Ki-67 proliferation index was approximately 2-4% in the most proliferative areas. Based on histomorphologic and immunohistochemical findings, a final diagnosis of nodular fasciitis was made. The representative microscopic photographs and Immunohistochemistry have been demonstrated in Fig 4 and Fig 5, respectively. The patient was extubated on the 10th postoperative day. She has shown progressive recovery, weight gain, and has been regularly followed up in the ENT & Pediatric OPD for the six months without any evidence of recurrence.

## Discussion

Nodular fasciitis (NF) is a self-limiting, reactive, proliferative tumor-like lesion characterized by excessive growth of fibroblasts and myofibroblasts (2). It is most commonly found in the upper extremities, followed by the trunk, and less commonly in the head and neck, including rare sites like the eyelids, face, neck, and oral cavity. Additionally, NF has been reported in atypical locations, including the bladder, mammary glands, and vocal cords. Very few cases have been documented involving the airway (3), and to date, tracheal involvement presenting with stridor has yet to be reported. 

The pathogenesis of NF remains uncertain, though prior trauma is associated with 5-10% of cases, suggesting a hyper-responsive mechanism that triggers increased mitotic activity in specific individuals (4). Diagnosing NF is generally straightforward when characteristic histological features are present in common anatomical locations. However, clinical misdiagnosis is more likely when NF occurs in atypical or rare sites. In this case, the main clinical symptoms included dysphonia, dyspnea, and foreign body sensation. The patient also presented with stridor and severe respiratory distress, which progressed into a life-threatening medical emergency as the mass was nearly obstructed in the lower trachea. Making a definitive airway and clinical diagnosis is quite challenging in pediatrics within the specified time encountered in the present case. In the present case, we secured the airway by doing a tracheotomy, although we did not put the tracheostomy tube, as there was very minimal space surrounding the mass. Rather, a low tracheotomy was performed, keeping in mind the distal location of the mass. The removal of the mass was quite challenging, as we had to intervene very quickly because of poor pulmonary function due to recurrent pneumonia. The mass was excised through the tracheotomy under endoscopic visualization with the help of coblation. 

A similar case was documented by Yusuf Shieba et al., in which the mass was almost occluding the trachea, and a rigid bronchoscopy was performed for diagnosis (3). In contrast, there was not much time to diagnose the tracheal mass in the preoperative period, as there was severe stridor. Although diagnosis usually depends on clinical manifestations, in this case, it wasn't easy, as we didn't have time for bronchoscopy or radiological evaluation of the airway. The final diagnosis mainly depended upon the histopathology, as observed in this case. Histopathological examination showed a significant proliferation of fibroblasts within a mucoid matrix, localized capillary hyperplasia, a characteristic ribbon-like or vortex collagen fiber arrangement, deep nuclear staining, and prominent nucleoli (5). 

Immunohistochemically, NF typically shows strong positivity for SMA and vimentin, with negativity for markers such as S-100 protein, desmin, and CD34, which helps differentiate NF from malignant spindle cell neoplasms, such as sarcomatous lesions (4,6). 

Differential diagnoses for benign spindle cell tumors include fibrous histiocytoma, pyogenic granuloma, postoperative spindle cell nodule, myofibroma, and peripheral nerve tumor, while malignancies to be ruled out include Kaposi sarcoma, spindle cell carcinoma, and spindle cell melanoma (7). 

As a benign hyperplastic lesion, NF generally requires only local surgical resection, with no need for radical surgery. Regular follow-up is essential as spontaneous resolution can occur within 4 to 8 weeks (8,9). Surgical excision is the established mode of treatment of NF, and some surgeons prefer to give perilesional corticosteroid injection as an add-on therapy (1). As demonstrated in the present case, we completely excised with coblation. Although the relapse rate is less than 10%, it is still unpredictable, especially if there is subtotal resection of the lesion. It is assumed that there is increased proliferation of fibroblasts, myxoid matrix, and infiltration of inflammatory cells, triggered by surgical manipulation (10). In the present case, the patient remains asymptomatic at 12 months of follow-up, without recurrence of the disease, which may be due to the complete resection of the tumor.

## Conclusion

NF is a rare airway tumor that usually occurs in adults and can present with pneumonia-like symptoms. NF of the trachea in a child presenting as a life-threatening obstruction of the airway is extremely rare in clinical practice. Early resuscitation is essential to save the patient's life, and histopathology confirms the diagnosis.
